# Awareness of Human Papillomavirus and Its Oncogenic Potential in Head and Neck Cancer among Students: Still More Questions than Answers

**DOI:** 10.3390/ijerph17228667

**Published:** 2020-11-22

**Authors:** Joanna Jeruzal-Świątecka, Wioletta Pietruszewska

**Affiliations:** Department of Otolaryngology, Head and Neck Oncology, Medical University of Lodz, 90-419 Lodz, Poland; joanna.jeruzal@gmail.com

**Keywords:** human papilloma virus, cancer, awareness, head and neck squamous cell carcinoma, student, survey study

## Abstract

In the past years, human papilloma virus (HPV) has been proved to be an important risk factor for head and neck squamous cell carcinomas (HNSCCs), especially in the oropharynx (OPCCS). The aim of this study was to assess the level of knowledge about HPV among students and to raise their awareness on the issue. A 22-question questionnaire was uploaded to an online service. Information about the project was sent out to students from three Universities in Lodz, Poland. All data were collected via questionnaire website tools. A total of 1710 students participated in this study. The group was divided into medical (MS) and non-medical (non-MS) students. Merely 59.38% of the non-MS had ever heard about HPV. Only 44.74% of the non-MS knew about vaccines against HPV. The oncogenic potential of HPV was evident for 81.17% of the MS and only 55.92% of the non-MS. Very similar numbers of respondents from both groups (39.21% vs. 36.47%) knew that HPV may cause cancers other than cervical. Nearly half of the respondents from both groups (46.28% vs. 48.32%) did not know about the risk of developing oral or oropharyngeal cancer. The level of knowledge about the consequences of HPV infection in head and neck cancers in young adults remains insufficient.

## 1. Introduction

Health education is one of the most important elements affecting public health. Creating habits conducive to maintaining individual and community health should become a basic element of social education. Health campaigns focused on cancer prevention are being conducted all over Europe. They mainly refer to the most prevalent cancers, such as lung, breast, cervical and colorectal cancers or melanoma. Some of the cancer-inducing agents have been identified. Notably, it has been proved that certain chronic viral infections may lead to the development of cancer [1]. Approximately 15% of cancers diagnosed in 2012 were attributed to carcinogenic infections, such as Helicobacter pylori, human papillomavirus (HPV), hepatitis B virus, hepatitis C virus and Epstein–Barr virus infections [1].

HPV is a DNA virus from the papillomavirus family, from which approximately 200 types have already been identified. HPV viruses have been divided into two groups according to their oncogenic potential: high and low risk. Some estimations suggest that 80% of sexually active women will be exposed to HPV during their lifetime. High-risk types, HPV-16 and HPV-18 in particular, are associated with more than 80% of cervical cancers, but the infection can also result in carcinomas of the vulva, vagina, penis and anus [2]. Even though substantial effort has been made over the last decade to raise public awareness of the relationship between HPV infection and cervical cancer, the latest research shows that over the past years, HPV infections have become an important risk factor for a number of head and neck squamous cell carcinomas (HNSCCs). They seem to be a particularly significant factor in oropharyngeal squamous cell carcinomas (OPCCS) situated in lingual, palatine, and tonsillar regions. HPV-dependent tumors account for 4–61% of oral cancers, 15–100% of middle throat cancers and up to 54% of larynx tumors [3]. HNSCC remains a major cause of morbidity and mortality and the sixth most common cancer type in the world. It accounts for 9% of all cancers in men and 5% of cancers in women [4]. Until recently, the most important risk factors for the development of head and neck tumors were smoking and the consumption of hard alcohol. However, the latest research suggests that HPV has become the most important risk factor for cancer in this area in the past 20 years. Although the incidence of oral cancer remains stable, the number of tonsillar cancer cases has been increasing annually. According to GLOBCAN statistics [4], more than 92 thousand new cases of oropharyngeal cancer were diagnosed in 2018, and HPV DNA was found in 35–50% of the cases. HPV spreads predominantly through oral–oral, oral–genital and genital–genital sexual contact. It manifests an affinity to the epithelial cells including the non-keratinized stratified squamous epithelium of the mouth, throat, esophagus, vagina, cervix and anus, as well as the keratinized stratified squamous epithelium lining the outer surface of the body [5]. 

Raising awareness of the potential consequences resulting from HPV infections seems to be a crucial element of health promotion and anti-cancer prophylaxis. The aim of this survey study conducted among students was to assess their current level of knowledge about HPV and enhance their understanding of the issue.

## 2. Materials and Methods 

A questionnaire consisting of 22 questions was created and uploaded to the SurveyMonkey online questionnaires website. Information about the project was sent out directly to students from three Universities in Lodz by e-mail, and posted on social media and student organizations’ websites. The survey was titled “Could kissing harm your health? Test your knowledge of Human Papilloma Virus (HPV)”. 

The first six questions gathered information on metrics such as age, gender and the participant’s place of origin. In Question 5, students had to indicate the field of their studies, choosing from humanistic fields (e.g., philology, law and administration, economics, management, sociology, and finance and banking), technical fields (e.g., biotechnology, mechatronics, IT, engineering, construction, architecture and mechanics), medical fields (e.g., pharmacy, nursing, medicine, dentistry, veterinary medicine and physiotherapy) and nature fields (e.g., biology, agriculture and forestry). For the purpose of statistical analysis, the respondents were then divided into two groups: medical and non-medical students.

The next two questions referred to different diseases that can be transferred via droplets and/or saliva. The main focus of the questionnaire was the second part, which referred to the human papilloma virus—it verified the participants’ knowledge about its variations, route of infection, oncogenic potential and vaccination programs. For educational purposes, a short informative note enclosing all the most important information on the subject was presented at the end of the survey. The questionnaire construct itself divided respondents into two groups. If the participant was not aware of the existence of human papilloma virus, she/he would finish the questionnaire on Question 9 and go straight to the information sheet at the end.

The last two questions were available to the respondents who marked “oral cancer”, “oropharyngeal cancer” or “all” in Question 20. Question 21 referred to the risk factors for developing oral and oropharyngeal cancer. The last question assessed if the knowledge about HPV influenced the sexual behavior of the respondents. Statistical analysis was conducted by descriptive statistical methods using the data generated from SurveyMonkey website statistics. Full questionnaire in Polish and English is available as a Supplementary Materials.

All subjects gave their informed consent for inclusion before they participated in the study. The study was conducted in accordance with the Declaration of Helsinki, and the protocol was approved by the Bioethics Committee of the Medical University of Lodz (RNN/338/19/KE). 

## 3. Results

**Figure 1:**
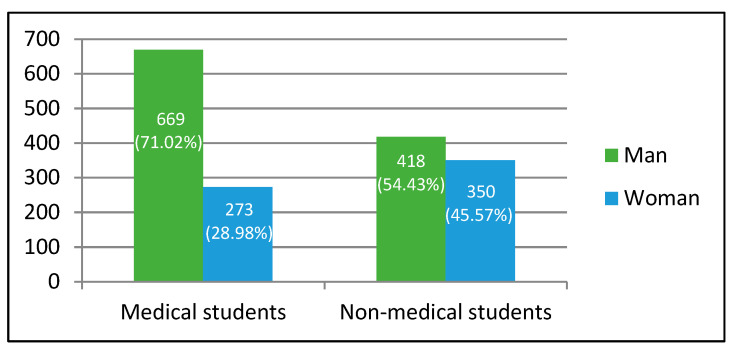
Gender distribution of the human papilloma virus (HPV) knowledge survey respondents, with a division into medical and non-medical students (N = 1710).

A total of 1710 students participated in the study. The majority were male (63.64%), and the most represented age group was those between 20 and 29 years of age. Medical students accounted for approximately 55% of the respondents; non-medical students, for 45% (Figure 1).

**Figure 2:**
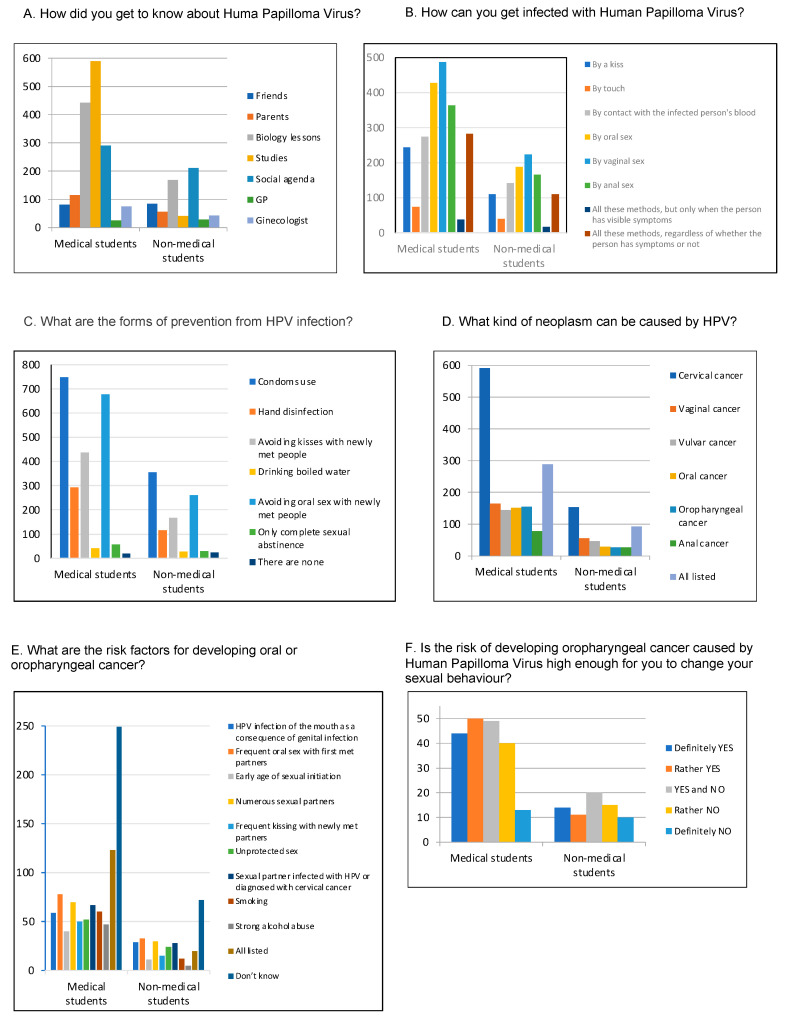
Statistical distribution of respondents’ answers to selected questions from HPV knowledge survey, with the division into medical and non-medical students (N = 1710).

Merely 59.38% of the non-medical students had ever heard about human papilloma virus, while virtually all the medical students (96.39%) were already aware of its existence (Table 1). Those respondents (N = 1270) mostly owed their knowledge to biology classes, social agenda and studies (Figure 2A). Approximately 96.48% of the medical students and 86.4% of the non-medical students acknowledging the existence of HPV were also aware of HPV’s infectious potential (Table 1). Only 44.74% of the non-medical students were aware of the existence of a vaccine against HPV, in contrast to more than 70% of the respondents of the medical student group (Table 1). HPV was described as a cause of diseases by 78.29% and 91.19% of the non-medical and medical students, respectively (Table 1). When asked about the available ways of preventing HPV infections, both groups indicated the most important factors: the use of condoms and avoiding kissing and oral sex with newly met people. The oncogenic potential of HPV was evident for 81.17% of the medical students and for over 50% of the non-medical students (55.92%) (Table 1).

Cervical cancer was the HPV-related neoplasm most commonly indicated by both groups. Nevertheless, nearly the same percentages of respondents from both groups (39.21% of the medical students; 36.47% of the non-medical students) knew that HPV may cause all the neoplasms listed in the survey (cervical, vaginal, vulvar, oral, oropharyngeal and anal cancer). (Figure 2D). To clarify the level of knowledge concerning head and neck neoplasms, all those respondents were asked a question about the risk factors for developing oral or oropharyngeal cancer. As a result, almost half of both groups (46.28% of the medical students; 48.32% of the non-medical students) turned out to be ignorant about the subject (Figure 2E). Surprisingly, there was no significant difference between the medical and non-medical students in regard to this question. In the last question, the participants were asked if the knowledge of HPV’s infectious and carcinogenic potential might influence their sexual behavior. The answers varied and were spread out almost uniformly for all the possible options, ranging from “definitely yes” to “rather no”, with the smallest percentage for “definitely no” (Figure 2F).

## 4. Discussion

Until recently, the development of head and neck cancers was attributed mainly to factors such as smoking and strong alcohol consumption. However, recent research indicates that over the past 20 years, HPV has become one of the most important risk factors in HNSCC. The importance of the virus in the etiopathogenesis of oropharyngeal cancers has to be emphasized, as the number of HPV-positive cancers in this area increased by over 225% [6]. A meta-analysis of contemporary studies on oropharyngeal cancer estimates a 69.7% prevalence of HPV association in HNSCC patients in North America and 73.1% in Europe [7]. It predominantly affects men between 35 and 70 years of age. Knowledge and awareness of the threat favors an early implementation of preventive behaviors in order to reduce incidence rates. There is also a difference in the course and prognosis of HPV-positive patients in comparison to those of HPV-negative patients suffering from oropharyngeal cancer. HPV-positive oropharyngeal cancer seems to be more responsive to chemotherapy and radiation than HPV-negative disease, giving a much better prognosis of recovery [8,9,10].

Recent studies have shown a strong association of a higher number of lifetime sexual partners, higher number of oral sex partners, earlier age at sexual initiation and history of same-sex sexual contact with HPV-positive oropharyngeal cancer groups as compared to patients who were HPV negative [11,12].

The purpose of this work was to determine the level of knowledge about HPV among young people, their awareness of the threat caused by infection and the available prophylactic methods.

In our study, 1364 people out of 1710 respondents (79.44%) had heard about the HPV virus before (Table 1). According to Blake et al.’s [13] study, out of 3185 Americans surveyed, approximately 68% had heard of HPV. Those with a high school degree were less likely to acquire that knowledge than those with a college education (OR= 0.58, 95% CI=0.34, 0.98). In their study, only 33.2% of the respondents were college graduates or postgraduates. In our study, we wanted to investigate not only whether the degree of education mattered, but also if the type of studies was relevant. That is why we compared the answers given by non-medical and medical students. It has been shown that students of medical faculties were more likely to demonstrate prior knowledge about HPV. Respondents aware of the existence of the virus, regardless of the field of study, recognized equally well its infectious potential (MS: 96.48% vs. NMS: 86.40%) (Table 1).

The existing literature supports a predominantly sexual means of transmission for oral HPV infection. Vaginal, anal and oral sex and open mouth kissing have all been linked to an increased risk of HPV infection [14,15]. A meta-analysis of studies from 1980 to 1998 assessing HPV in oral tissues and cells reported that the likelihood of detecting HPV in the normal oral mucosa was 10.0%. This was significantly less than in benign leucoplakia (22.2%), intraepithelial neoplasia (26.2%), verrucous carcinoma (29.5%) and oral squamous cell carcinoma (46.5%), but nevertheless, it was detected even in perfectly normal oral mucosa [16]. A large number of saliva samples from the general public, which would include exfoliated epithelial cells, have been screened across the USA, and a 3.1% carriage of high-risk HPV genotypes was reported [17]. Regardless of the field of study, our respondents were able to precisely indicate the possible transmission paths for the virus (Figure 2B).

It has been demonstrated that barrier contraceptive methods reduce the risk of genital HPV infection as well as other STIs, and they are the main form of infection prevention according to the literature [18,19]. Our respondents, when asked about the form of infection prevention, most frequently noted using condoms (MS: 85.39% vs. NMS: 90.1%), avoiding kissing (MS: 49.89% vs. NMS: 42.39%) and avoiding oral sex (MS: 77.28% vs. NMS: 66.24%) with a newly met person. In this question, the type of studies also seemed to be irrelevant (Figure 2C).

The quality of oral hygiene seems to corelate with oral and oropharyngeal cancer, but there is little evidence of its possible correlation with oral HPV infections [20,21]. Bui et al. defined a significant association between self-reported poor oral health and a higher incidence of oral HPV infection [22]. More objective research was conducted by Torre et al., who used the approximal plaque index (API), the gingival bleeding index (GBI) and the lifetime number of extracted teeth as more unbiased indicators [23]. They were determined in 187 patients, and the presence of oral low-risk and/or high-risk HPV was investigated by brush smear testing. Thirty-nine participants had positive oral HPV tests (27 high-risk HPV, 26 low-risk HPV, and 14 low- and high-risk HPV). A higher API, higher GBI and greater number of extracted teeth were significantly correlated with the presence of high-risk HPV. The presence of low-risk HPV was significantly higher in patients with an API > 40% and GBI > 40% (OR: 7.89). This confirms a relationship between the quality of oral hygiene, determined by objective markers, and indicates the need to also pay attention to oral hygiene education.

The ACIP (Advisory Committee on Immunization Practices) recommends routine HPV vaccination at the age of 11 or 12 years, but it can be given as early as at the age of 9. The ACIP also recommends vaccination for females until 26 years of age and for males until 21 years of age in the case of individuals who were not adequately vaccinated previously. Males are recommended to be vaccinated if aged 22 through 26 years, especially in the case of men who have sex with men [24]. Though the recommendations are clear, the vaccination coverage rates are still very low. Thirty-seven of the 53 countries in Europe have introduced the vaccine into their national routine immunization schedules and are vaccinating girls. However, several countries, such as Austria, Australia and the United States of America, also offer the vaccine to boys to ensure their direct and immediate protection from genital warts and forms of HPV-related cancer that affect both men and women [25]. In Poland, HPV vaccination is not included in the mandatory vaccination schedule and can only be administrated on a voluntary basis. The full cost of two or three doses of this vaccination has to be entirely covered by parents. Even though the HPV vaccine has been available on the Polish market for almost 20 years, HPV vaccination coverage in adolescent girls is estimated at 1.5–10% and is much lower than that reported in countries where the cost of vaccinations is covered by the national budget [26]. According to the latest publications, as a result of a vaccination program implemented in Australia, by the year 2028, annually, fewer than four women in every 100,000 are expected to be diagnosed with cervical cancer, effectively eliminating the disease as a public health problem [27]. Out of the 1364 students who knew about the existence of the HPV virus, only 850 people knew about the vaccine (62.32%) (Table 1). Mostly, they were medical students (76%). HPV vaccines are highly effective and safe and are a powerful prevention tool for reducing HPV infections and HPV-associated cancers [28,29,30]. These data confirm the continuous necessity of educating young people, who in the future, will decide on the vaccination protocol for their children.

The majority of the medical students were also aware of the oncogenic potential of the HPV virus, but most of them associated it exclusively with cervical cancer (Table 1 and Figure 2D). That is similar to what McBride et al. showed in their study, where the knowledge of the link between HPV and cancers other than cervical cancer (anal, penile and oral) was universally low in both men and women [31]. Only 31.50% of male and 28.75% of female respondents knew about its association with oral cancer.

Taberna et al. [32], in their study, were trying to determine changes in sexual behavior after a diagnosis of HPV-positive and HPV-negative oral cancer, surveying patients and their partners. The data suggest little difference in relationship stress or sexual behaviors subsequent to a diagnosis of HPV-positive or HPV-negative OSCC. Sexual behavior changed significantly in the 6 months after a diagnosis of OSCC, regardless of tumor HPV status. This was true, despite the fact that patients and partners generally reported high-quality relationships and a greater appreciation of their partner. Approximately one-half of the participants in the study expressed concern about HPV transmission through oral and vaginal sex, but few expressed concerns about transmission through kissing or nonsexual contact. In our study, the respondents did not show significant consideration in terms of changing their behavior due to the risk of developing oropharyngeal cancer caused by HPV infection (Figure 2F).

A very important element of our questionnaire was the final information page that every respondent could read after filling out the survey. The text about the facts concerning HPV was very short and concise. It was created with an intention to help to impart knowledge in the case of respondents with low awareness and to extend and consolidate information for those who demonstrated considerable knowledge on the subject. Willey et al. [33] designed an educational module consisting of a 1-hour lecture, a case presentation, assigned articles, 90 min wrap-up quizzes, a group clinical application exercise, and a 20 min lecture on a case and real-world applications for first-year medical students. A pre-/post-test survey was performed on general knowledge of HPV, satisfaction with education and willingness to recommend vaccination for HPV, which indicated that they significantly improved their knowledge of HPV (from 66.3% pre-module to 86.3% post-module, *p* < 0.001) and their satisfaction with medical education on vaccination-related topics, as well as increasing their willingness to recommend vaccination (from 58% pre-module to 100% post-module, *p* < 0.001). There is no doubt that these types of educational activities should be scheduled in all types of universities or even at the high school education level. Our research shows that a group of students of medical faculties show much greater knowledge about the HPV virus; therefore, focusing on the other groups should produce even more significant results.

Due to the specific selection of the study groups, the conclusions of this work cannot be applied to the general academic community. When assessing the knowledge of medical students, the year of the studies should be taken into account because the knowledge about the HPV virus and its oncological potential may depend on whether the students have already completed clinical classes in the Gynecology and ENT departments. Additionally, family history should be considered in both groups. It can be assumed that the appearance of HPV-positive cancer in the family could create a need to pursue this topic and thus broaden one’s knowledge to a degree much greater than that of an average student. Nevertheless, the results show visible trends that could be applied to these groups and indicate the direction for further research on a larger scale.

## 5. Conclusions

In recent years, the knowledge about the HPV virus, its infectivity and the possible consequences of chronic infection has increased significantly. To date, public awareness of the influence of HPV infection as a risk factor has been restricted to cervical cancer exclusively. The level of knowledge about the possible consequences of virus infection in the context of head and neck cancers remains insufficient. The fact that almost all the medical students knew about the existence of the HPV virus and yet their detailed knowledge on the subject did not differ significantly compared to the group of non-medical students is perplexing. For several years, the importance of HPV infection in the etiopathogenesis of oral and oropharyngeal cancer has been underlined, but it still seems to be overlooked even in medical education. The awareness of the threat and the knowledge about the possible prophylactic methods seem to be crucial, especially among young people, who can protect themselves from infection by following prevention principles and may also protect their children by applying protective vaccinations in the future.

## Figures and Tables

**Table 1:** Statistical distribution of respondents’ answers to selected questions from HPV knowledge survey with division into medical and non-medical students.

QUESTION	ANSWER	Medical Students		Non-Medical Students	
		N	%	N	%
Have you ever heard about Human Papilloma Virus (HPV)?	YES	908	96.39%	456	59.38%
	NO	34	3.61%	312	40.63%
	Σ	942		768	
Can you get infected with HPV?	YES	876	96.48%	394	86.40%
	NO	9	0.99%	4	0.88%
	DON’T KNOW	23	2.53%	58	12.72%
Are there any vaccines against Human Papilloma Virus (HPV)?	YES	646	71.15%	204	44.74%
	NO	61	6.72%	41	8.99%
	DON’T KNOW	201	22.14%	211	46.27%
Can Human Papilloma Virus (HPV) cause a disease?	YES	828	91.19%	357	78.29%
	NO	10	1.10%	10	2.19%
	DON’T KNOW	70	7.71%	89	19.52%
Can human papilloma virus (HPV) infection lead to cancer?	YES	737	81.17%	255	55.92%
	NO	17	1.87%	23	5.04%
	DON’T KNOW	154	16.96%	178	39.04%
	Σ	908		456	

## Supplementary Materials

The following are available online at https://www.mdpi.com/1660-4601/17/22/8667/s1: File S1. Questionnaire for this research.
