# Supplementary material for: Awareness of Human Papillomavirus and Its Oncogenic Potential in Head and Neck Cancer among Students: Still More Questions than Answers
Source: Int J Environ Res Public Health. 2020 Nov 22;17(22):8667. doi: 10.3390/ijerph17228667 (PMC7700204; doi:10.3390/ijerph17228667)
Supplement: Supplementary file 1 [file ijerph-17-08667-s001.pdf]

English translation

By completing this survey, you consent to the processing and use of personal data in accordance with the Act of May 10, 2018 on the protection of personal data (GDPR) in connection with the processing of personal data to the extent specified in art. 2 and art. 3 of Regulation (EU) 2016/679 of the European Parliament and of the Council of 27 April 2016 on the protection of individuals with regard to the processing of personal data and on the free movement of such data, and repealing Directive 95/46 / EC (Journal of Laws of the EU 2016, p. 1) while maintaining anonymity.

Can a simple kiss be harmful?
Test your knowledge about Human Papilloma Virus (HPV).

1. Gender
- ☐ woman
- ☐ man

2. Age
- ☐ 16-19
- ☐ 20-25
- ☐ 26-29
- ☐ 30 and more

3. The size of the town of your origin
- ☐ village
- ☐ City of up to 50,000 residents
- ☐ City 50-100 thousand inhabitants
- ☐ City of 100-500 thousand inhabitants
- ☐ City over 500,000 inhabitants

4. At what stage of education are you currently?
- ☐ High school
- ☐ Bachelor studies
- ☐ Engineering studies
- ☐ Masters studies

Commented [JJ1]: If the answer is "high school" participant omits question 5, other answers go straight to question 6

5. In what field are your studies?
- ☐ Humanistic fields (e.g.: philology, law and administration, economics, management, sociology, finance and banking)
- ☐ Technical fields (e.g.: biotechnology, mechatronics, IT, engineering, construction, architecture, mechanics)
- ☐ Medical fields (e.g.: pharmacy, nursing, medicine, dentistry, veterinary medicine, physiotherapy)
- ☐ Nature fields (e.g.: biology, agriculture, forestry)

6. What secondary school do you attend?
- ☐ High school

- ☐ Profiled high school
- ☐ Technical school
- ☐ Basic profession school

7. Do you think that you can get an infection while kissing?
    - ☐ Yes
    - ☐ No
    - ☐ I do not know

Commented [JJ2]: If the answer is NO or DON'T KNOW participant goes to question 9

8. What kind of infection you can get during a kiss?
- ☐ Herpes
- ☐ Influenza
- ☐ Mononucleosis
- ☐ HPV
- ☐ Angina
- ☐ Hepatitis A virus
- ☐ Mycosis of the mouth
- ☐ Other, what? ...
- ☐ All listed

9. Have you ever heard about human papilloma virus (HPV; Human Papilloma Virus)?
- ☐ Yes
- ☐ No

Commented [JJ3]: If NO participant finishes the questionnai

10. How did you get to know about Human Papilloma Virus (HPV)?
    - ☐ friends
    - ☐ parents
    - ☐ biology lessons in school
    - ☐ studies
    - ☐ social campaigns
    - ☐ GP
    - ☐ gynaecologist
    - ☐ other sources, which? ...

11. Can human papillomavirus (HPV) be infectious?
    - ☐ Yes
    - ☐ No
    - ☐ I do not know

Commented [JJ4]: If the answer is NO or DON'T KNOW participant goes to question 15

12. How can you get infected with human papilloma virus (HPV)?
- ☐ By a kiss
- ☐ By touch
- ☐ By contact with the infected person's blood
- ☐ By oral sex

- ☐ By vaginal sex
- ☐ By anal sex
- ☐ Other, what? ...
- ☐ All these methods, but only when the person has visible symptoms
- ☐ All these methods, regardless of whether the person has symptoms or not

13. Who, in your opinion, is more exposed to human papilloma virus (HPV) infection?
    - ☐ A young person often changing partners
    - ☐ A mature person in a stable relationship

14. What are the forms of prevention from human papilloma virus (HPV) infection?
    - ☐ Condoms use
    - ☐ Hand disinfection
    - ☐ Avoiding kisses with newly met people
    - ☐ Drinking boiled water
    - ☐ Avoiding oral sex with a newly-met person
    - ☐ Only complete sexual abstinence
    - ☐ There are not ways of prevencion
    - ☐ Other, what? ...

15. Are there any vaccines against human papilloma virus (HPV)?
    - ☐ Yes
    - ☐ No
    - ☐ I do not know

Commented [JJ5]: If the answer is NO or DON'T KNOW participant omits question 16

16. Who should be vaccinated against human papilloma virus (HPV)?
    - ☐ Girls before sexual initiation
    - ☐ Boys before sexual initiation
    - ☐ Boys and girls before sexual initiation
    - ☐ I do not know

17. Can human papilloma virus (HPV) infection cause a disease?
    - ☐ Yes
    - ☐ No
    - ☐ I do not know

Commented [JJ6]: If the answer is NO or DON'T KNOW participant goes to question 19

18. Which diseases can be caused by HPV infection?
    - ☐ Genital warts
    - ☐ Recurrent respiratory/ laryngeal papillomatosis
    - ☐ Skin warts
    - ☐ Papillomas of the mouth and throat
    - ☐ Other, what? ...
    - ☐ All listed

19. Can human papilloma virus (HPV) infection lead to cancer?
    - ☐ Yes
    - ☐ No

Commented [JJ7]: If NO or DON'T KNOW participant finishe the questionnaire

- I do not know

20. What neoplasm can be caused by human papilloma virus (HPV) infection?
- Cervical cancer
- Vaginal cancer
- Vulvar cancer
- Oral cancer
- Oropharyngeal cancer
- Anus cancer
- Other, what? ...
- All listed
- I do not know

Commented [JJ8]: If participant doesn't mark oral cancer, oropharyngeal cancer or all she/he finishes questionnaire

21. What are the risk factors for developing oral or oropharyngeal cancer?
- HPV infection of the mouth as a consequence of genital infection
- Oral sex with a large number of newly met partners
- Early sexual initiation
- A large number of sexual partners
- Frequent kisses with newly met people
- No condom use
- Sexual partner infected with HPV or suffering from cervical cancer
- Smoking
- Hard alcohol abuse
- All listed
- I do not know

22. Do you think that the risk of oral and oropharyngeal cancer associated with human papilloma virus (HPV) infection is so high that it may be affecting your sexual behaviour?
- Definitely yes
- Rather yes
- Neither yes nor no
- Probably not
- Definitely not

Wypełniając ankietę wyrażają Państwo zgodę na przetwarzanie i wykorzystanie danych osobowych zgodnie z ustawą z dnia 10 maja 2018 roku o ochronie danych osobowych (RODO) w związku z przetwarzaniem danych osobowych w zakresie określonym w art. 2 i art. 3 rozporządzenia Parlamentu Europejskiego i Rady (UE) 2016/679 z dnia 27 kwietnia 2016 r. w sprawie ochrony osób fizycznych w związku z przetwarzaniem danych osobowych i w sprawie swobodnego przepływu takich danych oraz uchylenia dyrektywy 95/46/WE (Dz. Urz. UE L 119 z 04.05.2016, str. 1) z zachowaniem anonimowości.

Czy pocałunki mogą być szkodliwe?

Sprawdź swoją wiedzę na temat wirusa brodawczaka ludzkiego (HPV; Human Papilloma Virus).

1. Płeć
   - kobieta,
   - mężczyzna
2. Wiek
   - 16-19
   - 20-25
   - 26-29
   - 30 i więcej
3. Wielkość miejscowości z której pochodzisz
   - Wieś
   - Miasto do 50 tyś mieszkańców
   - Miasto 50-100 tyś mieszkańców
   - Miasto 100-500 tyś mieszkańców
   - Miasto powyżej 500 tyś mieszkańców
4. Na jakim etapie edukacji jesteś obecnie?
   - Szkoła średnia
   - Studia licencjackie
   - Studia inżynierskie
   - Studia magisterskie
5. Na jakim kierunku studiujesz?
   - **Kierunki humanistyczne** (np. : filologia, prawo i administracja, ekonomia, zarządzanie, socjologia, finanse i bankowość)
   - **Kierunki techniczne** (np. : biotechnologia, mechatronika, informatyka, inżynieria, budownictwo, architektura, mechanika)
   - **Kierunki medyczne** (np. : farmacja, pielęgniarstwo, lekarski, stomatologia, weterynaria, fizjoterapia)
   - **Kierunki przyrodnicze** (np. : biologia, rolnictwo, leśnictwo)
6. Do jakiej szkoły średniej uczęszczasz?
   - Liceum ogólnokształcące
   - Liceum profilowane
   - Technikum
   - Zasadnicza szkoła zawodowa

Commented [A9]: Jeżeli szkoła średnia pomija pyt 5 i odpow[illegible] na pyt 6, jeżeli reszta to pomija pyt 6

7. Czy uważasz, że podczas pocałunku można się czymś zarazić?

- Tak
- Nie
- Nie wiem

8. Czym możesz się zarazić w trakcie pocałunku?

- Opryszczką
- Grypą
- Mononukleozą zakaźną
- Wirusem HPV
- Anginą
- Wirusem zapalenia wątroby typu A
- Grzybicą jamy ustnej
- Inne, jakie?...
- Wszystkie wymienione

9. Czy słyszałeś o wirusie brodawczaka ludzkiego (HPV; Human Papilloma Virus)?

- Tak
- Nie

10. W jaki sposób dowiedziałeś się o wirusie brodawczaka ludzkiego (HPV; Human Papilloma Virus)?

- od znajomych
- od rodziców
- z lekcji biologii w szkole średniej
- ze studiów
- z akcji społecznych
- od lekarza rodzinnego
- od ginekologa
- z innych źródeł, jakich?...

11. Czy wirusem brodawczaka ludzkiego (HPV; Human Papilloma Virus) można się zarazić?

- Tak
- Nie
- Nie wiem

12. W jaki sposób można się zarazić wirusem brodawczaka ludzkiego (HPV; Human Papilloma Virus)?

- Przez pocałunek
- Przez dotyk

Commented [A10]: Jeżeli nie lub nie wiem przechodzisz do p

Commented [A11]: Jeżeli odpowiedź nie to koniec ankiety, j
tak to ankieta dalej normalnie

Commented [A12]: Jeżeli odpowiedź nie lub nie wiem to idz
do pyt 15

- ☐ Przez kontakt z krwią osoby zakażonej
- ☐ Przez seks oralny
- ☐ Przez seks waginalny
- ☐ Przez seks analny
- ☐ Inne, jakie?...
- ☐ Wszystkimi tymi sposobami, ale tylko gdy osoba ma widoczne objawy
- ☐ Wszystkimi tymi sposobami niezależnie czy osoba ma objawy czy nie

13. Kto według Ciebie jest bardziej narażony na zakażenie wirusem brodawczaka ludzkiego (HPV; Human Papilloma Virus)?

- ☐ Młoda osoba często zmieniająca partnerki/partnerów
- ☐ Dojrzała osoba w stałym związku

14. Jakie są formy prewencji zakażenia wirusem brodawczaka ludzkiego (HPV; Human Papilloma Virus)?

- ☐ Stosowanie prezerwatyw
- ☐ Dezynfekcja dłoni
- ☐ Unikanie pocałunków z nowo poznanymi osobami
- ☐ Picie przegotowanej wody
- ☐ Unikanie sexu oralnego z nowo poznaną osobą
- ☐ Tylko zupełna abstynencja seksualna
- ☐ Nie ma żadnych
- ☐ Inne, jakie?...

15. Czy istnieją szczepionki przeciwko wirusowi brodawczaka ludzkiego (HPV; Human Papilloma Virus)?

Commented [A13]: Jeżeli odpowiedź nie lub nie wiem to bez[illegible] 16

- ☐ Tak
- ☐ Nie
- ☐ Nie wiem

16. Kogo powinno się szczepić przeciwko wirusowi brodawczaka ludzkiego (HPV; Human Papilloma Virus)?

- ☐ Dziewczynki przed inicjacją seksualną
- ☐ Chłopców przed inicjacją seksualną
- ☐ Chłopców i dziewczynki przed inicjacją seksualną
- ☐ Nie wiem

17. Czy zakażenie wirusem brodawczaka ludzkiego (HPV; Human Papilloma Virus) może prowadzić do rozwoju choroby?

Commented [A14]: Jeżeli nie lub nie wiem idzie do pyt 19

- ☐ Tak
- ☐ Nie
- ☐ Nie wiem

18. Jakie choroby może wywołać zakażenie HPV?

- Kłykciny kończyste
- Nawracająca brodawczakowatość układu oddechowego
- Kurzajki
- Brodawczaki jamy ustnej i gardła
- Inne, jakie?...
- Wszystkie wymienione

19. Czy zakażenie wirusem brodawczaka ludzkiego (HPV; Human Papilloma Virus) może prowadzić do rozwoju nowotworu?

- Tak
- Nie
- Nie wiem

Commented [A15]: Jeżeli odpowiedź nie lub nie wiem to kon[illegible] ankiety

20. Jakie nowotwory może wywołać zakażenie wirusem brodawczaka ludzkiego (HPV; Human Papilloma Virus)?

- Rak szyjki macicy
- Rak pochwy
- Rak sromu
- Nowotwory jamy ustnej
- Nowotwory ustnej część gardła
- Rak odbytu
- Inne, jakie?...
- Wszystkie wymienione
- Nie wiem

Commented [A16]: Tylko jeżeli odpowiedź wszystkie, odpowiedz nowotwory jamy ustnej lub nowotwory ustnej części gardła to dalsze dwa pytania, w innym przypadku koniec ankiety

21. Co toruje drogę rozwojowi nowotworów jamy ustnej i gardła?

- Zakażenie wirusem HPV jamy ustnej w konsekwencji zakażenia narządów płciowych
- Seks oralny z dużą liczbą nowo poznanych partnerów/ partnerek
- Obniżony wiek inicjacji seksualnej
- Duża liczba partnerów/ partnerek seksualnych
- Duża liczba pocałunków z nowo poznanymi osobami
- Brak stosowania prezerwatyw
- Partnerka seksualna zakażona wirusem HPV lub cierpiąca na raka szyjki macicy
- Palenie tytoniu
- Nadużywanie alkoholu wysokoprocentowego
- Wszystkie wymienione
- Nie wiem

22. Czy uważasz że ryzyko zachorowania na nowotwór jamy ustnej lub gardła związane z zakażeniem wirusem brodawczaka ludzkiego (HPV; Human Papilloma Virus) w trakcie pocałunku i/lub sexu oralnego jest na tyle wysokie że wpływa na Twoje zachowania seksualne?

- Zdecydowanie tak

- ☐ Raczej tak
- ☐ Ani tak, ani nie
- ☐ Raczej nie
- ☐ Zdecydowanie nie
